# Nicotine Component of Cigarette Smoke Extract (CSE) Decreases the Cytotoxicity of CSE in BEAS-2B Cells Stably Expressing Human Cytochrome P450 2A13

**DOI:** 10.3390/ijerph14101221

**Published:** 2017-10-13

**Authors:** Minghui Ji, Yudong Zhang, Na Li, Chao Wang, Rong Xia, Zhan Zhang, Shou-Lin Wang

**Affiliations:** 1 Key Lab of Modern Toxicology of Ministry of Education, School of Public Health, Nanjing Medical University, 101 Longmian Avenue, Nanjing 211166, China; jiminghui77@sina.com (M.J.); 18262637913@126.com (Y.Z.); dyyflina@163.com (N.L.); chaowang_sph_njmu@163.com (C.W.); xrong1008@163.com (R.X.); zhanzhang@njmu.edu.cn (Z.Z.); 2 State Key Lab of Reproductive Medicine, Institute of Toxicology, Nanjing Medical University, 101 Longmian Avenue, Nanjing 211166, China; 3 School of Nursing, Nanjing Medical University, 101 Longmian Avenue, Nanjing 211166, China

**Keywords:** cigarette smoke extract, nicotine, cytochrome P450 2A13, cytotoxicity, BEAS-2B cells

## Abstract

Cytochrome P450 2A13 (CYP2A13), an extrahepatic enzyme mainly expressed in the human respiratory system, has been reported to mediate the metabolism and toxicity of cigarette smoke. We previously found that nicotine inhibited 4-(methylnitrosamino)-1-(3-pyridyl)-1-butanone (NNK) metabolism by CYP2A13, but its influence on other components of cigarette smoke remains unclear. The nicotine component of cigarette smoke extract (CSE) was separated, purified, and identified using high-performance liquid chromatography (HPLC) and ultra-performance liquid chromatography tandem mass spectrometry (UPLC-MS/MS), splitting CSE into a nicotine section (CSE-N) and nicotine-free section (CSE-O). Cell viability and apoptosis by Cell Counting Kit-8 (CCK-8) and flow cytometry assays were conducted on immortalized human bronchial epithelial (BEAS-2B) cells stably expressing CYP2A13 (B-2A13) or vector (B-V), respectively. Interestingly, CSE and CSE-O were toxic to BEAS-2B cells whereas CSE-N showed less cytotoxicity. CSE-O was more toxic to B-2A13 cells than to B-V cells (IC_50_ of 2.49% vs. 7.06%), which was flatted by 8-methoxypsoralen (8-MOP), a CYP inhibitor. CSE-O rather than CSE or CSE-N increased apoptosis of B-2A13 cells rather than B-V cells. Accordingly, compared to CSE-N and CSE, CSE-O significantly changed the expression of three pairs of pro- and anti-apoptotic proteins, Bcl-2 Associated X Protein/B cell lymphoma-2 (Bax/Bcl-2), Cleaved Poly (Adenosine Diphosphate-Ribose) Polymerase/Poly (Adenosine Diphosphate-Ribose) Polymerase (C-PARP/PARP), and C-caspase-3/caspase-3, in B-2A13 cells. In addition, recombination of CSE-N and CSE-O (CSE-O/N) showed similar cytotoxicity and apoptosis to the original CSE. These results demonstrate that the nicotine component decreases the metabolic activation of CYP2A13 to CSE and aids in understanding the critical role of CYP2A13 in human respiratory diseases caused by cigarette smoking.

## 1. Introduction

Tobacco use is a serious threat to health, and there are approximately 1.1 billion smokers worldwide [1]. China has a large market for tobacco consumption, as it produces and consumes approximately 40% of the world’s cigarettes [2]. Cigarette smoke is the most common exogenous hazard for humans. It causes cancer in multiple organs and is the major cause of lung cancer [3]. Tobacco use causes approximately 6.3 million deaths every year [4], and approximately 0.45 million cancer cases per year are caused by smoking [5]. When tobacco is burned during smoking, many reaction products are formed. Among the approximately 5000 types of compounds observed in cigarette smoke [6], more than 60 have been identified as carcinogenic, including polycyclic aromatic hydrocarbons (PAHs), tobacco-specific N-nitrosamines [7] and aromatic amines [8]. It is well known that tobacco smoke is a major component of air pollution. Exposure to environmental tobacco smoke (ETS) is prevalent worldwide despite growing awareness of its adverse health effects on non-smokers, especially for children. ETS contains the same toxic substances as identified in mainstream tobacco smoke; exposure to ETS leads to reduced lung function, increased risk of respiratory tract illnesses, increased prevalence of non-allergic bronchial hyperresponsiveness, and possibly increased risk of asthma [9].

Typically, many components of cigarette smoke are pre-carcinogens that must be activated by phase I metabolic enzymes (mainly cytochrome P450 enzymes—CYP) to exert their carcinogenic effects [10,11]. CYP2A13 is an extrahepatic metabolic enzyme that is mainly expressed in the human respiratory system, particularly in the trachea and bronchial epithelial cells [12]. Previous studies reported that a variant genotype of CYP2A13, A257C, showed a low metabolic activity and was associated with a substantially reduced risk of lung adenocarcinoma [13,14]. There are several substrates of CYP2A13 in cigarette smoke, such as nicotine, 4-(methylnitrosamino)-1-(3-pyridyl)-1-butanone (NNK), benzo-pyrene, naphthalene, and 3-methylindole (3-MI) [15]. NNK is the most well-known carcinogen in cigarette smoke, and CYP2A13 was reported to play a key role in NNK-mediated lung cancer [16,17]. PAHs in cigarette smoke, such as pyrene, 1-hydroxypyrene, 1-nitropyrene and 1-acetylpyrene, can be metabolically activated by CYP2A13 and are thought to be associated with lung cancer [18]. Heterocyclic amines constitute another type of substrate of CYP2A13. For example, it has been reported that 2-amino-3-methylimiidazo [4,5-f] quinolone (IQ) may lead to lung cancer in mice [19].

It should be noted that some of the non-carcinogenic substrates activated by CYP2A13 might influence the activities of carcinogens during cigarette smoking, particularly the abundant compounds such as nicotine. Nicotine is the most abundant alkaloid of tobacco, comprising approximately 95% of the total alkaloid content. Nicotine exerts its addictive effects by stimulating nicotinic acetylcholine receptors located in the central nervous system. At a high alkaline pH, nicotine is in a non-ionized state and can be readily absorbed across the epithelium of the lung, oral mucosa, nose, and skin [20]. It undergoes metabolism by CYP2A13 and CYP2A6 in the liver and lung to generate the metabolite cotinine [21].

Generally, the toxicity of nicotine and its metabolites mediated by CYP2A13 is relatively low [22], and CYP2A13 is inactivated during the metabolism of nicotine [23]. Our previous study showed that high concentrations of nicotine diminished the CYP2A13-induced metabolic activity of aflatoxin B1 (AFB_1_) and NNK [22], suggesting that nicotine in cigarette smoke might inhibit the metabolic activation of CYP2A13 to the components that occur in low contents in cigarette smoke. Therefore, removing nicotine in cigarette smoke would help to illustrate the role of CYP2A13 in cigarette smoking-induced toxic effects (especially the indirect carcinogens) of lung tissues.

In the present study, cigarette smoke extract (CSE) was prepared and separated into two parts, nicotine section (CSE-N) and nicotine-free section (CSE-O), using high-performance liquid chromatography (HPLC) and ultra-performance liquid chromatography tandem mass spectrometry (UPLC-MS/MS). Immortalized human bronchial epithelial (BEAS-2B) cells that stably express human CYP2A13 (B-2A13) and vector (B-V), as previously established and successfully used in our lab [22], were used to compare the cytotoxicity induced by CSE, CSE-N, and CSE-O. The study aims to understand the role of CYP2A13 in the metabolic activation of toxic components of cigarettes and related respiratory diseases, including lung cancer.

## 2. Materials and Methods

### 2.1. Cigarettes, Chemical and Reagents

The cigarettes used in the study were Kentucky reference cigarettes 3R4F from the University of Kentucky (Lexington, KY, USA), containing 9.4 mg of tar and 0.726 mg of nicotine per cigarette; (−) nicotine standard was obtained from Accustandard (New Haven, CT, USA). HPLC-grade methanol, formic acid (FA), and ammonium acetate were purchased from Merck (Darmstadt, Germany); 8-methoxypsoralen (8-MOP) dissolved in dimethyl sulfoxide (DMSO) was obtained from Sigma-Aldrich (St. Louis, MO, USA). Dulbecco’s modified Eagle’s medium (DMEM) was purchased from GIBCO-BRL (Grand Island, NY, USA). Antibodies specific to caspase-3, C-caspase-3, Poly (Adenosine Diphosphate–Ribose) Polymerase (PARP), Cleaved Poly (Adenosine Diphosphate–Ribose) Polymerase (C-PARP), Bcl-2 Associated X Protein (Bax) and B cell lymphoma-2 (Bcl-2) were purchased from Cell Signaling Technology (Danvers, MA, USA). Glyceraldehyde 3-phosphate dehydrogenase (GAPDH) antibody, penicillin, streptomycin, and cell counting kit 8 (CCK-8) were purchased from Beyotime (Shanghai, China).

### 2.2. Preparation of CSE and Separation of Nicotine from CSE

CSE was prepared following the method described previously [24]. Briefly, one cigarette with a filter was fixed horizontally to be burned, and the main stream of smoke was aspirated at a flow rate of 75 mL/min (one cigarette per 5 min). Ten cigarettes were passed through a solvent of 10 mL to collect CSE, and this content was defined as 100% (one cigarette per 1 mL). In the pilot study, dichloromethane was selected as the best of the four candidate solvents that were tested (water, hexane, methanol, and dichloromethane) on the basis of the highest absorbance and absorption peak of the substances by HPLC (data not shown).

The HPLC system (LC-15C, Shimadzu, Kyoto, Japan) equipped with an ultraviolet (UV) detector and Welchrom-C18 (4.6 mm × 150 mm, 5 μm; Welch Material, Inc., Austin, TX, USA) was used for the determination and separation of nicotine from CSE. The detected condition for nicotine was as follows: column temperature: 25 °C; injection volume: 100 μL CSE or nicotine standard (400 μg/mL); wavelength: 260 nm. The mobile phase was composed of methanol and 0.1 M ammonium acetate. Following the peak and the retention time of the nicotine standard in HPLC, both CSE-N (time: 8.8–10.8 min) and CSE-O (time: 0–8.8 and 10.8–25 min) were collected, respectively. After this separation has occurred 10 times, the collected solution of CSE-N, CSE-O, and 1 mL of CSE in dichloromethane (equal to the amount of CSE from one cigarette) was freeze dried and then dissolved in 100 μL of DMSO as stock solution before use.

### 2.3. Identification of Nicotine by UPLC-MS/MS

Utimate 3000 UPLC coupled with a Q Exactive MS system (Thermo Fisher Scientific, Framingham, MA, USA) was used to identify the nicotine in CSE, CSE-N, and CSE-O in an optimized condition following the method described previously [25]. The UPLC system consisted of a vacuum degasser, a quaternary pump, an autosampler and a Hypersil GOLD C18 Column (100 mm × 2.1 mm, 1.8 μm; Thermo Fisher Scientific, Framingham, MA, USA). The column temperature was 35 °C, and the injection volume was 10 μL. The Q Exactive MS system was equipped with a quadrupole mass spectrometer and an obi-trap mass spectrometer. An electrospray ionization source (ESI) and full scan were used. The analysis of nicotine was carried out in the positive ionization mode with a source temperature of 350 °C and an ion source voltage of 3200 V.

### 2.4. Cell Viability Assay

BEAS-2B cells were originally purchased from ATCC (Manassas, VA, USA), and B-2A13 cells and B-V cells (control cells) had been previously established and successfully used in our lab [22]. B-2A13 cells and B-V cells were plated into 96-well plates (5000 cells/well) with 200 μL of growth medium and were cultured at 37 °C in 5% CO_2_ overnight. The cells were treated with CSE, CSE-N, CSE-O, a recombination of CSE-N and CSE-O (CSE-O/N) at different concentrations (0–10%), or nicotine standard (0–100 μM) for 24 h, and the same volume of DMSO (1%) was used as the control. In order to confirm the effects, the cells were co-treated with CSE, CSE-N, CSE-O at different concentrations and 8-MOP (1 μM; an inhibitor of CYP450s) for 24 h. After the treatments, cell viability was determined using the CCK-8 assay as described in our previous study [22].

### 2.5. Cell Apoptosis Analysis

After B-2A13 and B-V cells (control cells) were incubated in a 3.5 cm dish (2 × 10^5^ cells/well) at 37 °C in 5% CO_2_ overnight, they were treated with 4% CSE, 4% CSE-N, 4% CSE-O, CSE-O/N, or 100 μM nicotine standard for 24 h, and the same volume of DMSO (0.4%) was used as the control. Cell apoptosis was then analyzed using a flow cytometry assay, as described in our previous study [26].

### 2.6. Determination of the Expression of Apoptosis-Related Proteins in Cells

B-2A13 and B-V cells were plated into 10 cm plates (1 × 10^6^ cells/well) in the growth medium and cultured at 37 °C in 5% CO_2_ overnight. After treatment with 4% CSE, 4% CSE-N, 4% CSE-O, a recombination of 4% CSE-O/N, or 100 μM nicotine standard for 24 h, apoptosis-related proteins were examined by immunoblotting assays as described previously [22]. As is well known, caspase-3 is one of the key executioners of apoptosis, being responsible either partially or totally for the proteolytic cleavage of many key proteins, such as the nuclear enzyme poly polymerase (PARP). Cleaved caspase-3 (C-caspase-3) is an activated form of caspase-3 that acts as a lethal protease at the most distal stage of the apoptosis pathway. C-PARP is the cleavage of PARP, and it has been considered indicative of functional caspase activation. For the immunoblotting assay, cell lysates (50 μg) were separated by Sodium Dodecyl Sulfonate (SDS)-polyacrylamide gel electrophoresis and were transferred onto a polyvinylidene fluoride membrane (Millipore, Bedford, MA, USA). Using specific antibodies for caspase-3, C-caspase-3, Cleaved Poly (Adenosine Diphosphate–Ribose) Polymerase (C-PARP), Poly (Adenosine Diphosphate–Ribose) Polymerase (PARP), Bcl-2 Associated X Protein (Bax) or B cell lymphoma-2 (Bcl-2), the protein immune complexes were detected using an Electrochemiluminescence (ECL) immunoblotting assay kit. GAPDH was used as an internal control.

### 2.7. Statistical Analysis

The mean and standard deviation (SD) were calculated for all of the investigated parameters. The data were analyzed using SPSS 19.0 software for Windows (SPSS, Chicago, IL, USA). Significant differences between the treatment groups and controls were compared using one-way analysis of variance (ANOVA); *p* < 0.05 was considered statistically significant.

## 3. Results

### 3.1. Separation and Identification of Nicotine in CSE

**Figure 1:**
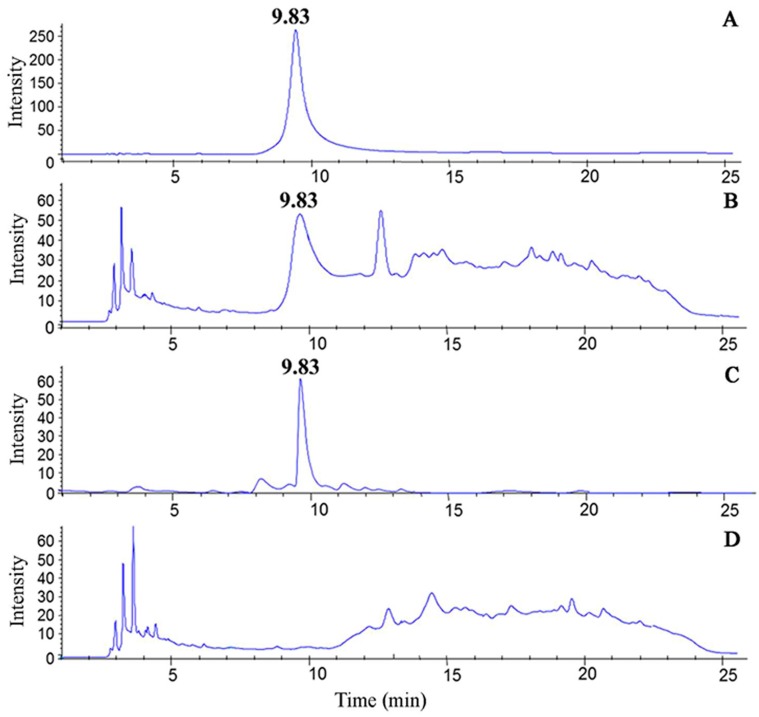
Chromatograms of nicotine in cigarette smoke extract (CSE) using high-performance liquid chromatography (HPLC). The retention time of nicotine occurs at 9.83 min. (**A**) Nicotine standard, (**B**) CSE, (**C**) nicotine section of CSE (CSE-N), and (**D**) nicotine-free section (CSE-O).

**Figure 2:**
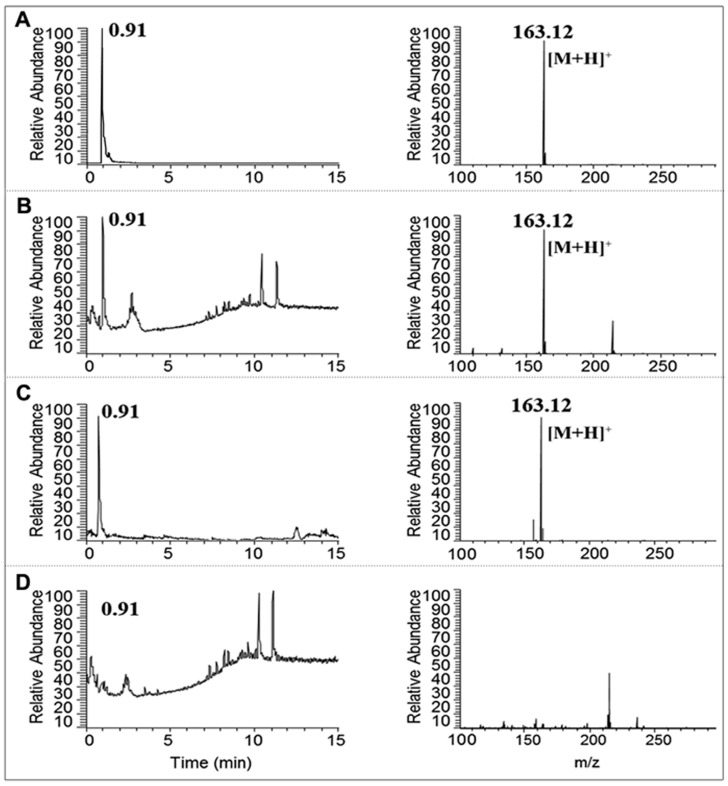
Chromatogram and mass spectra of nicotine in cigarette smoke extract (CSE) using ultra-performance liquid chromatography tandem mass spectrometry (UPLC-MS/MS). The retention time of nicotine occurs at 0.91 min (left panels), and *m*/*z* is 163.12 (right panels). (**A**) Nicotine standard, (**B**) CSE, (**C**) nicotine section of CSE (CSE-N), and (**D**) nicotine-free section (CSE-O).

A representative chromatogram of the nicotine standard is shown in Figure 1. The retention time of the peak was 9.83 min (8.8–10.8 min). Following the analysis of the total CSE, CSE was separated into two groups: the nicotine section (CSE-N; 8.8–10.8 min) and nicotine-free section (CSE-O; 0–8.8 and 10.8–25 min). Compared to the total CSE (Figure 1), CSE-N showed a sharp chromatogram peak at 9.83 min, but no similar peak was observed in CSE-O (Figure 1). CSE, CSE-N, and CSE-O were identified by UPLC-MS/MS. As shown in Figure 2, similarly to the standard nicotine, CSE and CSE-N were eluted at a retention time of 0.91 min and [M + H]^+^ *m*/*z* 163.12, and the concentration of nicotine was 103.66 ± 17.34 μg/cigarette. However, no chromatogram peak or mass spectra were observed in CSE-O (Figure 2), indicating that nicotine was completely separated from CSE.

### 3.2. Effects of Nicotine on CSE-Induced Cytotoxicity in B-2A13 Cells

**Figure 3:**
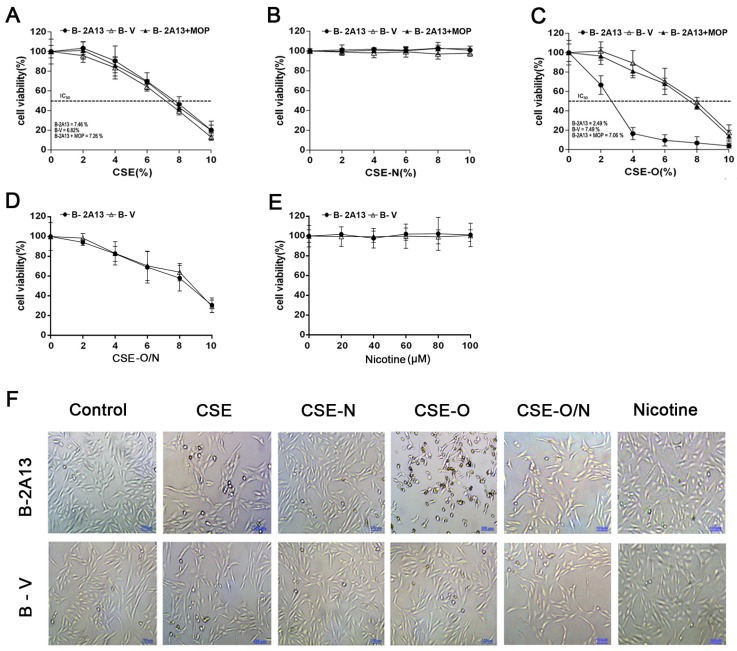
Cell viability of immortalized human bronchial epithelial (BEAS-2B) cells treated with cigarette smoke extract (CSE), nicotine section of CSE (CSE-N) and nicotine-free section of CSE (CSE-O). The cells were treated with CSE, CSE-N, CSE-O, CSE-O/N, or nicotine standard for 24 h, and a cell counting kit 8 (CCK-8) assay was used to determine cell viability. (**A**) CSE; (**B**) CSE-N; (**C**) CSE-O; (**D**) CSE-O/N; (**E**) nicotine standard; (**F**) representative photography of cells treated with 4% CSE, CSE-N, CSE-O, CSE-O/N, or nicotine standard (100 μM). The data are presented as the mean ± SD of three independent experiments with triplicate samples; bar: 50 μm.

The inhibitor of CYP enzymes 8-MOP [27] was used to demonstrate the effects of nicotine on the metabolic activation of CSE by CYP2A13. As shown in Figure 3, CSE induced a similar dose-dependent decrease in cell viability in B-V cells and B-2A13 cells and in B-2A13 cells co-treated with 8-MOP. CSE-N, similarly to the nicotine standard, seemed to have no cytotoxicity effects on the two cell types or on B-2A13 cells co-treated with 8-MOP. However, CSE-O induced significantly more cytotoxicity in B-2A13 cells than B-V cells (IC_50_ of 2.49% vs. 7.49%), and 8-MOP could reverse CSE-O-induced cell viability, similarly to B-V cells, in B-2A13 cells (IC_50_ = 7.06%). As expected, the cytotoxicity of CSE-O was decreased to that of the original CSE when it was recombined with CSE-N. These results are briefly summarized in Figure 3D and show that 4% CSE-O significantly decreased cell viability rates compared to 4% CSE or CSE-N in B-2A13 cells.

### 3.3. Effects of Nicotine on CSE-Induced Apoptosis and Expression of Related Proteins in B-2A13 Cells

**Figure 4:**
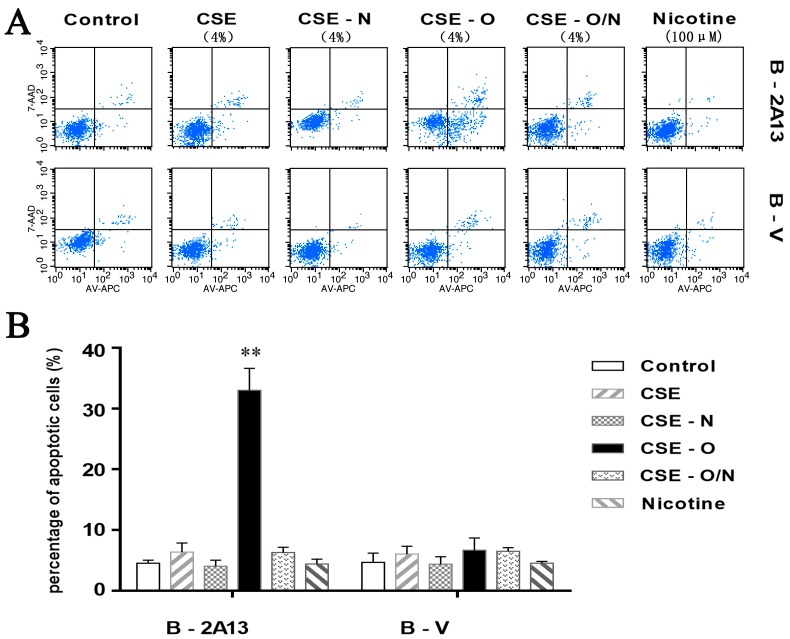
Cellular apoptosis of immortalized human bronchial epithelial (BEAS-2B) cells treated with cigarette smoke extract (CSE), nicotine section of CSE (CSE-N), and nicotine-free section of CSE (CSE-O). The cells were treated with 4% CSE, CSE-N, CSE-O, CSE-O/N, or nicotine standard for 24 h, and a flow cytometry assay was used to determine cellular apoptosis. (**A**) Spectrum, and (**B**) statistical data. The percentage of apoptotic cells is presented as the mean ± SD of three independent experiments with triplicate samples. ** *p* < 0.01, compared to cells treated with CSE or CSE-N.

**Figure 5:**
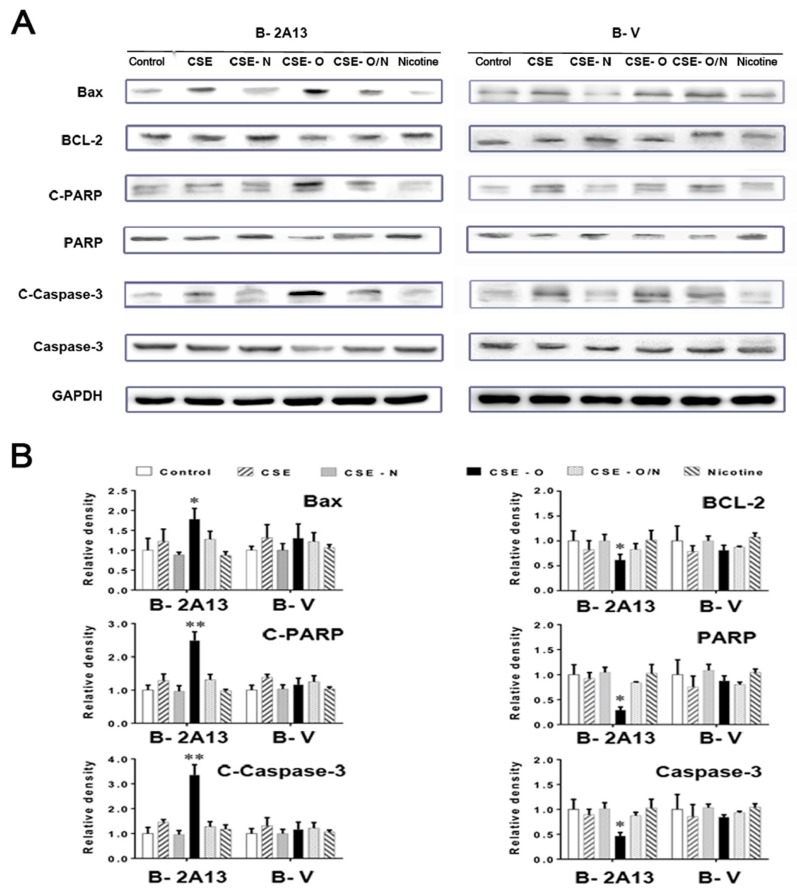
Expression of apoptosis-related proteins in immortalized human bronchial epithelial (BEAS-2B) cells treated with cigarette smoke extract (CSE), nicotine section of CSE (CSE-N) or nicotine-free section of CSE (CSE-O). The cells were treated with 4% CSE, CSE-N, CSE-O, CSE-O/N, or nicotine standard (100 μM) for 24 h. Cell lysates (50 μg) were prepared to determine the expression of apoptosis-related proteins by Western blot assay using antibodies specific for B cell lymphoma-2 (Bcl-2), Bcl-2 Associated X Protein (Bax), nuclear enzyme poly polymerase (PARP), cleaved PARP (C-PARP), caspase-3, and C-caspase-3. Glyceraldehyde 3-phosphate dehydrogenase (GAPDH) was used as an internal control. (**A**) Electrophoresis of Western blot. (**B**) Relative density scanned by Image J software using GAPDH as a reference. * *p* < 0.05; ** *p* < 0.01, compared to cells treated with CSE or CSE-N.

As shown in Figure 4, 4% CSE, CSE-N, CSE-O and nicotine standard showed low and similar apoptotic rates of B-V cells; however, 4% CSE-O significantly increased apoptosis (33%) compared to 4% CSE or CSE-N in B-2A13 cells. The apoptosis of CSE-O was decreased to that of the original CSE when it was recombined with CSE-N. The results were verified by the expression of apoptosis-related proteins. As shown in Figure 5, 4% CSE, CSE-N, CSE-O and nicotine standard showed similar changes in the protein expression in B-V cells. However, in B-2A13 cells, the expression of Bax, C-PARP, and C-caspase-3 significantly increased, whereas Bcl-2, PARP, and caspase-3 decreased following treatment with CSE-O compared to treatment with CSE or CSE-N. As expected, CSE, CSE-N and CSE-O/N showed similar profiles of protein expression in B-2A13 and B-V cells.

## 4. Discussion

Commonly, a Cambridge glass fiber filter and HPLC are used in the separation of CSE. A Cambridge glass fiber filter is used to remove nicotine from the mass stream of smoke being passed through, but the tar phase is always removed [28,29]. HPLC is commonly used in the separation and extraction of the components of traditional Chinese medicine [30]. HPLC can still guarantee the preparation of CSE with relatively simple, highly efficient, and real-time dynamic monitoring and easy-to-collect fractions [28]. HPLC not only removes nicotine from CSE but also collects the tar phase. Our study demonstrated that nicotine was completely removed from CSE and that a high purity of nicotine was collected (Figure 1). It was verified by UPLC-MS/MS that there was only one peak of nicotine (*m*/*z* 163.12) in CSE-N rather than in CSE (Figure 2), thus confirming the differences in composition between CSE-N and CSE-O in the present study.

There are thousands of compounds in cigarette smoke, dozens of which have been reported as substrates of CYP2A13 [31]. Because it is the major component of cigarette smoke and was reported to have a low cytotoxicity in our previous study [22], nicotine might obscure the potential toxicity of other active substrates of CSE in the presence of CYP2A13. Previous studies have shown that nicotine-free CSE decreases cell viability in mouse lung epithelium cell line LA-4 cells [32] and that the alteration of tobacco in an attempt to reduce cigarette nicotine content and attenuate addiction could result in increased toxicity and endothelial inflammatory response [33]. In the present study, CSE-O induced much greater cytotoxicity and apoptosis in BEAS-2B cells stably expressing CYP2A13 (B-2A13) than the total CSE induced, whereas CSE-N showed almost no toxicity in B-2A13 cells or B-V cells (control cells). In addition, 8-MOP, an inhibitor of cytochrome P450 enzymes [23,27] reversed CSE-O-induced the cytotoxicity of B-2A13 cells to that of the control cells, which indicated that nicotine inhibited the metabolic activation of CYP2A13 to CSE and that the removal of nicotine might cause greater damage to the respiratory system. As mentioned above, ETS contains the same toxic substances as are identified in mainstream tobacco smoke, and exposure to ETS has been shown to be associated with an increased prevalence of respiratory tract diseases [9]. Thus, it is possible that nicotine obscures the toxicity of other active components when humans are exposed to the mixtures in cigarette smoke, including ETS.

Members of the cytochrome P450 2A (CYP2A) subfamily are well known for their roles in the metabolism of nicotine and other precarcinogenic agents in tobacco, for example, NNK. CYP2A6 is mainly expressed in the human liver, whereas CYP2A13 is predominantly expressed in the respiratory tract. Although both the hepatic CYP2A6 and respiratory CYP2A13 metabolize the cigarette smoke-related compounds, CYP2A13 does so with much higher catalytic efficiency [34]. CYP2A13, in contrast to CYP2A6, plays an important role in tobacco-related tumorigenesis in the human respiratory tract [16,35,36]. In addition, X-ray structures of nicotine complexes with CYP2A13 and CYP2A6 yield a structural rationale for the preferential binding of nicotine to CYP2A13 [34]. Thus, among the components of cigarette smoke, nicotine, as a result of the dominant amount in CSE, might competitively consume CYP2A13 and subsequently reduce its metabolic activation of other active substrates, which was supported by our previous study that 100 μM of standard nicotine completely inhibited AFB1-induced cytotoxicity and apoptosis [22]. In the present study, the amount of nicotine per cigarette was 103.655 ± 17.336 μg, which was approximately 1/10 of the content of labeled nicotine (1.1 mg) in cigarettes because of the preparation of CSE from cigarettes with a filter. Thus, 4% CSE (containing 25.2 μM nicotine) induced much less cytotoxicity in B-2A13 cells compared to 4% CSE-O (Figure 3F). Further, this content of nicotine might consume CYP2A13 and subsequently reduce its activities [34]. In addition, nicotine itself has been reported to inhibit the activity of CYP enzymes. Previous studies have reported that nicotine was a more potent inhibitor of CYP 2E1 activity [37], and a series of 3-heteroaromatic analogues of nicotine were synthesized to delineate the structural and mechanistic requirements for selectively inhibiting CYP2A6 [38].

The commitment of a cell to enter a programmed cell death pathway represents a delicate balance between pro- and anti-apoptotic signals. The cell death induced by cigarette smoke might be caused by a mitochondria-mediated apoptotic pathway [39,40]. The Bcl-2 family is a key player in the mitochondria-mediated apoptotic pathway, and caspase-3 plays a critical role in executing the apoptotic process [41]. PARP is a caspase-3 substrate, and C-PARP is indicative of the activation of the apoptotic pathway. In the present study, the changes of three pairs of apoptosis-related proteins, Bcl-2/Bax, C-PARP/PARP, and C-caspase-3/caspase-3, were consistent with the cellular apoptosis presented in Figure 4. CYP2A13 was much more active during the metabolic activation of CSE-O in human lung epithelial cells, which suggests that CYP2A13 may be the primary metabolic enzyme involved in CSE-O metabolism in situ and possibly plays an important role in cigarette smoke-induced respiratory disorders.

The tissue- and cell-specific expression of CYP enzymes is believed to play a critical role in the metabolism in situ of various CYP substrates, particularly the precarcinogens in target tissues/cells. The dominant expression of CYP2A13 in human bronchial epithelium [12] and its high efficiency in the metabolic activation of tobacco-specific carcinogenic NNK are consistent with observations that most smoking-related lung cancers are bronchogenic [42]. In addition, 3-MI is also a preferential pneumotoxicant found in cigarette smoke. The metabolism of 3-MI by CYP2A13, but not hepatic CYPs, elicits equal or greater mutagenicity compared to prototypical cigarette smoke mutagens, for example, Benzopyrene, which suggests that 3-MI is a likely human pulmonary carcinogen [43]. Thus, in addition to the well-known NNK, other active substrates of CYP2A13 in CSE-O need to be explored in the future.

## 5. Conclusions

In summary, CYP2A13 is an enzyme that metabolizes several components of cigarette smoke in the respiratory system. The present study first demonstrated that nicotine in CSE decreased the metabolic activation of CYP2A13 to other toxic components of CSE, which should provide a novel basis that the cytochrome P450-mediated in situ metabolism might be helpful to identify the critical components of cigarette smoking, including ETS, in human respiratory diseases.

## Abbreviations

The following abbreviations are used in this manuscript.

CSE	Cigarette smoke extract
CYP	Cytochrome P450
CYP2A13	Cytochrome P450 2A13
NNK	4-(methylnitrosamino)-1-(3-pyridyl)-1-butanone
3-MI	3-methylindole
AFB1	Aflatoxin B1
BEAS-2B	Immortalized human bronchial epithelial (cells)
B-2A13	BEAS-2B cells stably expressing CYP2A13
HPLC	high-performance liquid chromatography
UPLC-MS/MS	Ultra-performance liquid chromatography tandem mass-spectrometry
C-PARP	Cleaved Poly (Adenosine Diphosphate-Ribose) Polymerase
PARP	Poly (Adenosine Diphosphate-Ribose) Polymerase
Bcl-2	B cell lymphoma-2
Bax	Bcl-2 Associated X Protein
